# Transmission of viral hepatitis through blood transfusion in Sweden, 1968 to 2012

**DOI:** 10.2807/1560-7917.ES.2020.25.29.1900537

**Published:** 2020-07-23

**Authors:** Viktor Dahl, Ammar Majeed, Agneta Wikman, Rut Norda, Gustaf Edgren

**Affiliations:** 1 Department of Public Health Analysis and Data Management, Public Health Agency of Sweden, Stockholm, Sweden; 2 Department of Medical Epidemiology and Biostatistics, Karolinska Institutet, Stockholm, Sweden; 3 Department of Gastroenterology, Alfred Health, Melbourne, Australia; 4 Central Clinical School, Monash University, Melbourne, Australia; 5 Department of Clinical Immunology and Transfusion Medicine, Karolinska University Hospital, Stockholm, Sweden; 6 Department of Laboratory Medicine, Karolinska Institutet, Stockholm, Sweden; 7 Department of Immunology, Genetics and Pathology, Uppsala University, Uppsala, Sweden; 8 Department of Medicine Solna, Clinical Epidemiology Division, Karolinska Institutet, Stockholm, Sweden; 9 Department of Cardiology, Södersjukhuset, Stockholm, Sweden

**Keywords:** blood, transfusion, viral, infection, hepatitis, Europe, Sweden

## Abstract

**Introduction:**

Viral hepatitis remains a significant threat to transfusion safety, although largely mitigated by donor screening.

**Aim:**

Our objective was to estimate the past and present burden of transfusion transmission of all types of viral hepatitis (A to E) and to find undiagnosed infections with hepatitis C virus (HCV).

**Method:**

We performed a retrospective cohort study using a database of the entire computerised transfusion experience of Sweden from 1968 to 2012 and linking it to a nationwide database of notifiable infections. We then used two independent statistical approaches. Firstly, we tracked recipients of blood from donors with confirmed viral hepatitis. Secondly, we computed a donor-specific risk score, defined as the difference between the observed and the expected number of HCV infections among all previous recipients of all donors, where thresholds were determined using simulation.

**Results:**

Among 1,146,307 transfused patients, more than 5,000 were infected with HCV. Transfusion transmission only occurred before 1992 when donor screening had been completely implemented. Overall, we found 44 donors and 1,180 recipients likely to be infected with HCV who were still alive but who remained undiagnosed.

**Conclusion:**

There is still a substantial number of individuals in Sweden who have probably been infected with HCV through blood transfusion and who are still unaware of their infection. We recommend that a follow-up study should be conducted to validate the method we used by approaching these individuals and offer testing. This would also serve as an opportunity to offer treatment to those who remain infected.

## Introduction

Although all of the hepatitis viruses (A to E) can be transmitted through blood transfusion, hepatitis B virus (HBV) and hepatitis C virus (HCV) have posed the most important threats to blood safety. This is due to their ability to cause chronic (often asymptomatic) infection associated with long-term complications such as cirrhosis of the liver and hepatocellular cancer. They are therefore associated with more morbidity and mortality than hepatitis A and E viruses (HAV and HEV) in high-income countries.

In Sweden, all forms of viral hepatitis fall under the communicable disease act and are notifiable by law. In 2018, 123 cases of HAV (61 of them infected in Sweden), 1,129 cases of HBV (54 of them infected in Sweden), 1,611 cases of HCV (882 of them infected in Sweden), 74 cases of HDV (three of them infected in Sweden) and 26 cases of HEV (18 cases infected in Sweden) were reported [1]. The notification system is designed to follow incidence but not prevalence; therefore, prevalence for the forms of viral hepatitis that can cause chronic infection is more uncertain as the infection can be cleared spontaneously or, for HCV, through treatment, or diagnosed individuals may migrate or die. An estimate by the public health agency of Sweden in 2015 was that 35,000–45,000 people in Sweden are living with diagnosed HCV; there is no similar estimate for HBV [2].

In high-income countries, transmission of viral hepatitis through blood transfusions has been minimised by the introduction of exclusion criteria for donors with high-risk behaviours or of markers for liver damage (e.g. elevated liver enzymes), and through the implementation of screening for both HBV and HCV. These preventive measures, which have been gradually implemented since the 1960s, have successfully reduced transmission rates of viral hepatitis to very low levels [3]. Through these screening efforts, and look-back studies in some countries, large numbers of donors and recipients of blood transfusions infected with HBV and HCV have been identified [4-6].

In Sweden, those who report clinical signs of hepatitis have been excluded from donating blood since 1968. This has been followed with specific testing for HBV, first with HBsAg screening which was implemented in 1970–72 and then with anti-HBc screening implemented in 1989–91. After the discovery of HCV, anti-HCV screening was added and fully implemented in 1992.

In 2019, Sweden is one of few high-income countries where HBV and HCV screening is still done using serological testing methods alone rather than with nucleic acid testing (NAT) alone or in combination with NAT. Because of the low prevalence of HBV and HCV infection this has been considered to be the most cost-effective approach [7]. To date there has not been a national look-back study to find those infected with HCV through blood transfusions before screening was implemented, but there were campaigns in 2008 urging those who received a transfusion before 1992 to undergo testing. However, we recently conducted a study on the possible transmission of neurodegenerative disease where we included HCV infection as a control since it is well known to be transmittable through transfusions; In that study, we found indications that there still are a substantial number of individuals alive who were infected with HCV through transfusions before 1992 who remain undiagnosed [8]. These findings were later repeated and confirmed in a study where we searched for unknown hepatitis viruses transmitted through transfusions [9].

The aim of this study was to estimate the incidence of viral hepatitis (A to E) transmitted through blood transfusions before and after screening was introduced for HCV, and to estimate the number of individuals who are likely to have been infected with HBV or HCV through blood transfusions, but who remain undiagnosed.

## Methods

With this in mind, we set up a nationwide retrospective look-back study based on the Scandinavian Donations and Transfusions (SCANDAT2) database which contains nationwide data on the identity of all blood donors and recipients of transfusion as well as the date of both donations and transfusions, and the national database for notifiable infections (SmiNet2) which, among other infectious diseases, contains the identity of the infected person and date of diagnosis for all types of viral hepatitis. 

### Study design

We designed the study as a retrospective cohort study where the cohort was defined as all individuals who had donated or received blood, as recorded in the Swedish part of the SCANDAT2 database. We then analysed the different types of viral hepatitis (A to E) separately. We stratified the analyses by calendar period of blood transfusion: 1968–1991 (little or no direct testing of blood donations for hepatitis C), 1992–1996 (testing of all blood donations for hepatitis C using first- or second-generation serology tests) and 1997–2012 (complete testing of all donations using the more sensitive third-generation serology tests).

### Data sources and record linkages

We based the study on three nationwide data sources. The first was the Swedish part of the SCANDAT2 database [10,11]. Among other variables it contains the unique national registration number (NRN) for all blood donors and recipients of transfusions and therefore allows identification of donors and their respective recipients [12]. Blood transfusion data are available for some of the Swedish counties from the late 1960s, with coverage gradually increasing to national completeness in 1996. The current version of the database contains blood donation and transfusion data until 2012. 

Using NRN, we linked data from SCANDAT2 to the national database for notifiable infections (SmiNet2), the second data source used [13]. This database is kept by the Public Health Agency of Sweden and contains data on all notifiable infections that fall under the communicable disease act, among them all types of viral hepatitis (A to E). Since Swedish law requires both the diagnosing clinicians and the microbiological laboratories to independently report all cases of notifiable diseases notifications, the sensitivity of the reporting system is estimated to be higher than 95% [14]. The SmiNet2 database contains data on several variables related to the notified case, e.g. date of diagnosis, county of residence, suspected country of infection and suspected mode of infection. The first electronic version of the surveillance system for notifiable infections (SmiNet) was introduced in 1997 and was then replaced by SmiNet2 in 2004–2006 which integrated the data from SmiNet [13].

We first extracted a complete list of NRN for the Swedish part of SCANDAT2 and generated a unique random identification number for each individual NRN, creating a key file coupling the random identification number to each NRN. We then transferred the key file to the Public Health Agency of Sweden where information on date and type of viral hepatitis (if any) was added to the key file. The notifications that lacked a full NRN were removed. We also added information extracted from a third data source, the Swedish population registry, on dates of death, emigration or removal from the population registry for other reasons. Then we removed the NRN and the key file was incorporated in a copy of SCANDAT2 where the NRN had also been removed, leaving only the random identification number for linkage. This generated a de-identified copy of SCANDAT2 with data on date and type of hepatitis diagnosis along with vital status and whether the person was still residing in Sweden or had emigrated.

### Statistical analysis

We used a similar two-pronged approach as in previous studies investigating transfusion-transmitted disease [8,9]. Firstly, we tested whether recipients from donors who were later diagnosed with viral hepatitis had an increased risk of being diagnosed with the same type of viral hepatitis. Secondly, because analyses based on the first approach are dependent on the donor being diagnosed with viral hepatitis, we also investigated whether we could identify donors with a higher incidence of viral hepatitis among their recipients than would be expected from chance alone. This second approach was based on a cumulative donor-specific disease excess score (DES) which is calculated as the difference between the observed and expected number of viral hepatitis diagnoses among all prior recipients of each donor. A DES value above 0 for a particular donor indicates that there have been more cases of viral hepatitis among past recipients of that particular donor than expected.

We calculated the DES separately for each type of hepatitis in a time-dependent fashion, so that the score was allowed to change with every donation. We calculated the observed count directly and the expected count by summing the predicted probabilities from a Cox regression model that incorporated each transfused unit as a separate entity, following the recipient for the occurrence of viral hepatitis from the date of transfusion until death or until end of follow-up (1 September 2017). These Cox models adjusted for the type of the transfused blood product (erythrocytes, platelets or plasma), hospital, calendar year of transfusion as well as age and sex of the transfused individual.

We then analysed in which date periods (i.e. 1968–1991, 1992–1996 or 1997–2012) we could detect transfusion transmission. To simplify these analyses, and to ensure that we only considered transfusions from one period at a time, we only assessed transfusions administered during the first 180 days following the first transfusion for each patient. We identified all blood units transfused during this period and tracked the contributing donor(s) and whether they were later diagnosed with viral hepatitis as well as the highest DES of all the blood units transfused during this period. For individuals who had received more than one unit from a donor who was later diagnosed with viral hepatitis, we used the unit with the shortest time from donation to hepatitis diagnosis as exposure point. We then fitted a Cox regression model following up all recipients for the diagnosis of viral hepatitis. The start of follow-up of the recipients was delayed by 180 days to avoid including patients in the analyses with a prevalent hepatitis infection already at the time of the transfusion. Individuals who died or were diagnosed with viral hepatitis before, or within 180 days of the first transfusion where thus excluded. The analyses were adjusted for county and year of transfusion (both as separate strata in a stratified Cox model), as well as sex (female or male), age (expressed as a restricted cubic spline with three evenly placed knots), number of transfusions (expressed as a cubic spline with seven knots manually placed at 1, 3, 5, 10, 20, 50 and 100 transfusions) and ABO blood group (A, AB, B or O). We conducted separate analyses for the first and second approaches. For the first approach, we assessed exposure both overall (i.e. whether the donor was ever diagnosed with hepatitis) and stratified by latency, i.e. time from donation to diagnosis of viral hepatitis in the donor (< 5, 5–10 and > 10 years).

Because the DES computations were based on tens of millions of statistical tests (one for each donation) we could expect many donors to achieve high DES values chance alone, even if there is no occurrence of transfusion transmission. We therefore set up a simulation to estimate what DES values would be outside the expected variation in a given year for each type of viral hepatitis. The simulation was based on our actual data but instead of using the observed events, we assigned events randomly using the expected probabilities and calculated a randomly generated, non-factual DES for each donation. We repeated this process 10,000 times with a variable random seed and used the results to compute the expected distribution of the DES metric. Because the maximum observed DES in a given year is likely to be influenced by both the number of donations and the implementation of screening tests, the distributions of the randomly generated maximum DES values were stratified by calendar year (Supplementary Figure 1). Using this distribution, we categorised all DES values as < 0, 0 to 2,5th percentile of maximum simulated value, 2.6th to 50th percentile, 51st to 97.5th percentile or > 97.5th percentile. Recognising that a donor who is both diagnosed with hepatitis and achieves a high DES is more likely to be infectious, we sub-divided the highest exposure category (i.e. > 97.5th percentile) based on the occurrence of hepatitis in the donor that contributed to the transfused unit with the highest risk.

We used the results from the above Cox regression analyses to investigate the transmission of viral hepatitis through blood transfusion during the three date periods and to identify donors and recipients who had a high probability of having been infected with viral hepatitis and who remained alive and undiagnosed at the end of follow-up. We assumed that donors and recipients had a high risk of being infected if they either donated or received a blood unit with a DES value that was higher than would be expected from chance alone, and where that DES category resulted in a statistically significantly increased risk in recipients in that date period. We also considered recipients to be infected if they had received a blood unit from a donor with a later viral hepatitis diagnosis in a date period when blood units of the same latency category resulted in statistically significantly increased risks. Here, we did not restrict the analyses to transfusions administered during the first 180 days of follow-up, but used instead the full follow-up of all patients.

For acute hepatitis (i.e. HAV and HEV), where there were very few events, we used a modified approach with a fixed 1-year latency, i.e. we considered as exposed patients who received a blood unit from a donor who was diagnosed within one year of donation. 

All data processing and statistical analyses were conducted using SAS statistical analysis software (Cary, NC), version 9.4.

### Ethical statement

The ethics review board in Stockholm, Sweden approved of the creation of the SCANDAT2 database and the conduct of this study (2017/1100–32).

## Results

In the results section we will focus on HCV and to a lesser extent HBV since these are the types of viral hepatitis where we found substantial evidence of transfusion transmission. A total of 1,146,307 transfused patients were included in the analysis of HCV transmission (Table 1). Patients receiving their first transfusion before 1992 were younger than those receiving their first transfusion in 1992–1996 or after 1996, with a median age of 61 years (interquartile range (IQR): 41–72) vs 70 years (IQR: 55–79) and 71 years (IQR: 55–80), respectively. As expected, length of follow-up was longer among those receiving their first transfusion in the period before 1992, with a median of 13.8 years (IQR: 4.4–28.3), compared with 8.7 years (IQR: 2.8–20.4) in 1992–1996 and 7.2 years (IQR: 2.7–11.6) after 1996.

**Table 1:** Characteristics of study population of blood transfusion recipients, analysis of transfusion-transmitted hepatitis, Sweden, 1968–2012 (n = 1,146,307)

	Patients transfused 1968–1991		Patients transfused 1992–1996		Patients transfused 1997–2012	
	n	%	n	%	n	%
Number of patients	291,790	25.5	178,443	15.6	676,074	59.0
Female	163,531	56.0	101,472	56.9	395,097	58.4
Median age at first transfusion in years (IQR)	61 (41–72)		70 (55–79)		71 (55–80)	
Median calendar year of first transfusion (IQR)	1983 (1978–1988)		1994 (1993–1995)		2004 (2000–2007)	
Median number of transfusions (IQR)	3 (2–6)		3 (2–5)		3 (2–5)	
Median duration of follow-up in years (IQR)	13.8 (4.4–28.3)		8.7 (2.8–20.4)		7.2 (2.7–11.6)	
Patients with < 5 years follow-up	79,032	27.1	64,398	36.1	250,900	37.1
Patients with 5–10 years follow-up	41,735	14.3	31,599	17.7	203,072	30.0
Patients with 10–20 years follow-up	56,762	19.5	35,549	19.9	219,496	32.5
Patients with > 20 years follow-up	114,261	39.2	46,897	26.3	2,606	0.4
Hepatitis occurrence in donor	6,120	2.1	731	0.4	914	0.14
Time from donation to hepatitis C occurrence in donor (years) with % of exposed recipients						
< 5 years from donation	416	6.8	214	29.3	364	39.8
5–9 years from donation	915	15.0	214	29.3	312	34.1
≥ 10 years from donation	4,789	78.3	303	41.5	238	26.0

IQR: interquartile range.

Table 2 presents analyses of the risk of infection with HCV in relation to donor HCV status, stratified by calendar period of first transfusion. Patients who received at least one transfusion from a donor with a subsequent diagnosis of HCV infection were at statistically significantly increased risk of HCV infection only for transfusions administered before 1992 (hazard ratio (HR) = 9.0; 95% confidence interval (CI): 8.1–10.0), but not in 1992–96 (HR = 1.3; 95% CI: 0.6–2.6) or after 1996 (HR = 2.0; 95% CI: 0.8–4.8). Although HR were consistently higher in all three periods when analyses were restricted to donors diagnosed with HCV infection within 5 years of donation, HR were only statistically significant in the first period (HR = 13.7; 95% CI: 10.3–18.0). For HBV, there were only two cases where both donor and recipient were diagnosed, both with the transfusion occurring before 1997, and accordingly, receipt of such a blood unit did not result in statistically significant excess risks (Supplementary Table 1).

**Table 2:** Relative risk of hepatitis C in relation to occurrence of the same disease in the contributing blood donor(s), overall and by latency in the donors, Sweden, 1968–2012 (n =1,146,307)

Disease	Donor diagnosed with hepatitis C		Donor not diagnosed with hepatitis C	
	Events/person-years	Hazard ratio (95% CI)	Events/person-years	Hazard ratio
**Patients transfused 1968–1991**				
All recipients transfused before 1992	557/86,599	9.0 (8.1–10.0)	2,476/4,812,308	1.00 (ref)
< 5 year latency in donor	54/4,370	13.7 (10.3–18.2)	2,476/4,812,308	1.00 (ref)
5–10 year latency in donor	96/11,151	10.5 (8.5–12.9)		
> 10 years latency in donor	407/71,078	8.4 (7.5–9.4)		
**Patients transfused 1992–1996**				
All recipients transfused 1992–1996	8/7,449	1.3 (0.6–2.6)	801/1,907,826	1.00 (ref)
< 5 year latency in donor	4/1,955	2.3 (0.9–6.2)	801/1,907,826	1.00 (ref)
5–10 year latency in donor	1/2,331	0.5 (0.1–3.8)		
> 10 years latency in donor	3/3,162	1.1 (0.4–3.5)		
**Patients transfused 1997–2012**				
All recipients transfused after 1996	5/7,511	2.0 (0.8–4.8)	1,297/5,182,445	1.00 (ref)
< 5 year latency in donor	3/3,001	3.0 (0.96–9.4)	1,297/5,182,445	1.00 (ref)
5–10 year latency in donor	2/2,420	2.5 (0.6–9.9)		
> 10 years latency in donor	0/2,090	0.0 (0.0–n.e.)		

CI: confidence interval; n.e.: not estimated; ref: reference value.

Numbers in the table may not add up because of rounding.

Analyses based on the DES metric for HCV are presented in Table 3 and for HBV in Supplementary Table 2. When DES values were categorised, using the simulated distribution, we detected a strong association between maximum donor DES and the risk of HCV infection in patients transfused before 1992. Here, patients who received a transfusion from a donor with a DES above the 97.5th percentile who was also diagnosed with HCV, resulted in a HR of 17.2 (95% CI: 10.6–27.9). Indeed, before 1992, all DES categories were statistically significantly associated with an increased risk of HCV (Table 3). Even recipients of units from donors with a DES above 0, but below the 2.5th percentile were at statistically significantly elevated risk of HCV, but with a considerably lesser risk magnitude (HR = 1.6; 95% CI: 1.4–1.8). After 1992, only patients who received a transfusion from a donor with a DES in the lowest risk strata (above 0 but below the 2.5th percentile) were at a statistically significantly, but still only modestly increased risk, with HR values of 1.2 (95% CI: 1.04–1.5) and 1.2 (95% CI: 1.1–1.4) from 1992 to 1996 and after 1996, respectively. For HBV, we only detected transfusion-transmission before 1992, with HR values of 4.2 (95% CI: 1.3–14.1) and 5.5 (95% CI: 1.3–23.1) among recipients of units from donors with a DES between the 2.5th and 50th and between the 50th and 97.5th percentile, respectively (Supplementary Table 2). There were no events in recipients of units from donors with DES for HBV above the 97.5th percentile.

**Table 3:** Relative risk of hepatitis C in relation to the maximum disease excess score among all contributing blood donors, stratified by calendar period of transfusion, Sweden, 1968–2012 (n = 1,146,307)

Maximum disease excess score, categorised by threshold from simulated distribution	1968–1991		1992–1996		After 1996	
	Events/person-years	Hazard ratio (95% CI)	Events/person-years	Hazard ratio (95% CI)	Events/person-years	Hazard ratio (95% CI)
< 0	457/1,320,108	1.0 (ref)	231/751,335	1.0 (ref)	556/2,681,582	1.0 (ref)
< 2.5th percentile	2,364/3,497,061	1.6 (1.4–1.8)	576/1,160,579	1.2 (1.04–1.5)	746/2,504,282	1.2 (1.1–1.4)
2.5th–50th percentile	95/34,608	4.9 (3.9–6.2)	1/2,783	0.4 (0.0–2.6)	0/3,295	0.0 (0.0–n.e.)
50th–97.5th percentile	76/31,652	4.1 (3.1–5.3)	1/578	1.4 (0.2–10.2)	0/797	0.0 (0.0–n.e.)
> 97.5th percentile + donor not diagnosed	23/13,395	4.7 (3.0–7.3)	0/0	0.0 (0.0–n.e.)	0/0	0.0 (0.0–n.e.)
97.5th percentile + donor diagnosed	18/2,084	17.2 (10.6–27.9)	0/0	0.0 (0.0–n.e.)	0/0	0.0 (0.0–n.e.)

CI: confidence interval; n.e.: not estimated; ref: reference value.

A total of 12,094 patients had received at least one transfusion from a donor in one of the strata in Tables 2 and 3 for which we saw substantial and statistically significant risk increases (i.e. any patients transfused before 1992 with units from a donor with a subsequent HCV diagnosis or a DES above the 2.5th percentile). Among those, 1,180 remained alive, lived in Sweden and were not diagnosed with HCV infection at the end of follow-up in 2017 (Table 4).

**Table 4:** Characteristics of patients at high risk of having been infected with hepatitis C virus by blood transfusion who remained alive and not diagnosed with hepatitis C at end of follow-up, Sweden, 1968–2012 (n = 1,180)

	n	%
S**ex**		
Female	700	59.3
Male	480	40.7
**Age at end of follow-up (years)**		
0–17	0	0.0
18–29	8	0.7
30–49	179	15.2
50–64	405	34.3
65–79	444	37.6
≥ 80	144	12.2
**Basis for risk assessment** ^a^		
Transfusion from donor diagnosed with hepatitis C		
< 5 year latency in donor	62	5.3
5–10 year latency in donor	143	12.1
> 10 years latency in donor	717	60.8
Transfusion from donor with elevated DES		
2.5th–50th percentile	152	12.9
50th–97.5th percentile	158	13.4
> 97.5th percentile	12	1.0

DES: disease excess score.

^a^ Numbers may add to more than 100% because some patients received blood units from more than one risk category.

Lastly, Table 5 presents data on the 871,793 contributing blood donors, in relation to their highest achieved DES percentile. Overall, the median highest achieved DES was 0 (IQR: 0–4.59) and the highest observed DES was 10.2 (11 observed vs 0.8 expected). The median DES was successively higher in each stratum (i.e. 2.5th–50th, 51st–97.5th and > 97.5th percentiles) as was the risk of HCV infection in the donors with successively higher HR of 67 (95% CI: 42–109), 99 (95% CI: 57–172) and 286 (95% CI: 127–645), respectively, compared with donors in the lowest stratum (Table 5). Among the 157 blood donors who ever achieved a DES above the simulated 2.5th percentile, 44 remained alive, lived in Sweden and were not diagnosed with HCV infection at end of follow-up.

**Table 5:** Characteristics of donors and risk of hepatitis, presented by disease excess score at last recorded donation, Sweden, 1968–2012 (n = 871,793)

	DES percentile at last recorded donation							
	< 2.5th percentile		2.5th–50th percentile		51st–97.5th percentile		> 97.5th percentile	
	n	%	n	%	n	%	n	%
Number of donors	871,637	100.0	95	0.01	54	0.01	8	0.00
Sex								
Female	389,687	44.7	9	9.5	5	9.3	0	0.0
Male	481,950	55.3	86	90.5	49	90.7	8	100.0
Calendar year of final donation								
1968–1991	288,159	33.1	77	81.1	50	92.6	8	100.0
1992–1996	167,362	19.2	15	15.8	1	1.9	0	0.0
1997–2012	416,116	47.7	3	3.2	3	5.6	0	0.0
Number of donations, median (IQR)	2 (2-4)		44 (24–80)		72 (43–109)		83 (42.5–102)	
Age at last donation in years, median (IQR)	30.9 (23.4–41.4)		44.6 (34.4–53.9)		45.0 (40.6–55.1)		48.0 (39.2–52.0)	
Final DES, median (min–max)	0.00 (0.00–4.59)		4.36 (1.00–5.73)		5.78 (1.94–8.03)		8.52 (2.92–10.2)	
Number of hepatitis C events	1,826	0.2	17	17.9	13	24.1	6	75.0
Hazard ratio of hepatitis (95% CI)^a^	1.00 (ref)		67 (42–109)		99 (57–172)		286 (127–645)	
Number of donors alive and undiagnosed at end of follow-up	707,060	81.1	31	32.6	13	24.1	0	0.0
Age at end of follow-up for those who remain alive and undiagnosed, in years, median (IQR)	51.9 (41.9–62.5)		71.3 (65.5–76.2)		76.0 (72.3–79.5)		NA	

CI: confidence interval; DES: disease excess score; IQR: interquartile range; NA: not applicable; ref: reference value.

^a^ Estimated using Cox regression adjusted for donor age, sex and calendar year.

For the other hepatitis viruses, there were 73 patients who received a blood unit from a donor who was diagnosed with HAV within 1 year of donating blood. One of these recipients was also diagnosed with HAV. As donor and recipient in this pair were diagnosed ca 1 and 2 months after donation/transfusion, respectively, this constitutes a possible case of HAV transmission. For HEV, there were four patients who received a blood unit from a donor who was diagnosed within 1 year of donating blood. None of these recipients developed HEV. Lastly, for HDV, there were three patients who received a blood unit from a donor with a subsequent HDV diagnosis. Of these, none were themselves diagnosed with HDV.

## Discussion

In this nationwide retrospective study based on national registers for both blood transfusions and notifiable infections and with data going back as far as the 1970s, with little or no loss of follow-up and near-complete ascertainment of all hepatitis diagnoses during this time period, we have performed a detailed characterisation of transmission of viral hepatitis between blood donors and recipients in Sweden.

The extent to which HAV and HEV, viruses that are mostly transmitted through the faecal-oral route and usually cause a transient acute infection, are a problem during transfusion has been discussed for some time. Several case reports on transfusion transmission of HAV from other countries show that transmission through transfusion is possible [15-18]. We found only one possible case of transmission of HAV where both the donor and the recipient were diagnosed with HAV infection after the blood donation. This indicates that in a country with low prevalence of HAV infection and with restrictions on blood donation after travelling abroad, HAV transmission through blood transfusion is very rare. Similarly, transmission of HEV through blood transfusion has been described both in countries with high incidence, such as India, and in countries with lower incidence such as France, Germany, Japan, Spain and the United Kingdom (UK) [19-23]. In 2017, screening for HEV among blood donors was introduced in two European Union countries (Ireland and the UK) [20]. While we did not find any case of suspected HEV transmission in Sweden, an earlier study found that one in 7,896 Swedish plasma donations were positive for HEV RNA, indicating that such transmission probably occurs also in Sweden [24]. However, because those infected with HEV do not always develop clinical disease [21] and because under-recognition of HEV infection among clinicians in Sweden is likely, we presume that our failure to detect any case of transmission of HEV was due to our approach which solely relied on diagnosed cases.

For HBV, we could not detect any signs of transmission through transfusions after 1992. In all, we found 11 individuals with a high risk of having been infected by HBV through a transfusion before 1992 who were still alive. For HCV we found ongoing transmission before anti-HCV screening had been fully introduced by 1992. This is in line with previous findings that, when screening started, 0.2–0.5% of Swedish blood donors were positive for anti-HCV [25,26]. In our analysis, we found 1,180 transfused patients and 44 blood donors – 1,224 individuals – who were deemed at a high risk of being infected with HCV and who remained alive, lived in Sweden and had not yet been diagnosed with HCV infection at the end of follow-up.

Although there have not been any nationwide look-back studies in Sweden, there has been some effort to find individuals who were infected with HCV through blood transfusion before screening was in place. In 2007, the National Board of Health and Welfare recommended that all Swedish counties offer HCV testing to those who, between 1965 and 1991, had received transfusion during neonatal care and to those who, during their childhood, had had heart surgery or had been treated for cancer. In 2010, this recommendation was extended to also include women who had received a transfusion during pregnancy or childhood during the same date period. Many of the Swedish counties attempted to conduct active case finding but were limited by the difficulties of finding information both on hepatitis diagnoses and on transfusions from the non-digitalised medical records of that era [27]. In the end, only five of 21 counties attempted active case finding. But all counties carried out an information campaign in 2008, urging those who had received a blood transfusion before 1992 to get tested for HCV. This effort resulted in the number of cases of HCV infection reported in 2008 going up to 2,526 from 2,134 in the previous year [1]. In one of the counties, an attempt was made to screen all people who had received a transfusion before 1992, resulting in the testing of 13,573 samples and a prevalence of 0.9% for anti-HCV and 0.8% for HCV RNA [28].

There are several limitations to our study. As it was based on notified infections only, the sensitivity was dependent on the extent to which infections had been diagnosed, which can also depend on whether the infection is symptomatic. This may have led to a reduced sensitivity to detect transmission. The sensitivity of our approach was also limited by the completeness of the data in the registers used; even if they have close to full completeness for recent data, the completeness for the years before the 1980s is likely to be lower. In addition, for donors achieving higher DES values, our method assumed a higher probability of being infected with viral hepatitis and transmitting it to their recipients. While this approach provides a novel way of identifying high-risk donors at population level, it has limited accuracy at an individual level. Donors with DES below 0 are assumed to have the lowest risk of transmitting viral hepatitis, but there might still be donors in this group who are infected with viral hepatitis but who still achieve only very low DES because they made only few donations. Such donors will therefore not be identified by our method, further reducing the sensitivity to detect transmission through transfusion.

## Conclusion

In this retrospective nationwide study investigating transmission of viral hepatitis through blood transfusion we found that such transmission decreased dramatically after screening for HCV was fully implemented in 1992. However, a total of 1,224 individuals likely to be infected with HCV remain alive, undiagnosed and live in Sweden. We recommend that a follow-up study should be conducted to validate the method we used by approaching these individuals to offer testing. This would also serve as an opportunity to offer treatment to those who remain infected.

## Supplementary Data

689000 Supplement
