# Supplementary material for: Transmission of viral hepatitis through blood transfusion in Sweden, 1968 to 2012
Source: Euro Surveill. 2020 Jul 23;25(29):1900537. doi: 10.2807/1560-7917.ES.2020.25.29.1900537 (PMC7384284; doi:10.2807/1560-7917.ES.2020.25.29.1900537)
Supplement: Supplement [file 19-00537_DAHL_Supplement.pdf]

Supplementary Material

This supplementary material is hosted by Eurosurveillance as supporting information alongside the article “Transmission of viral hepatitis through blood transfusion in Sweden between 1968 and 2012”, on behalf of the authors, who remain responsible for the accuracy and appropriateness of the content. The same standards for ethics, copyright, attributions and permissions as for the article apply. Supplements are not edited by Eurosurveillance and the journal is not responsible for the maintenance of any links or email addresses provided therein

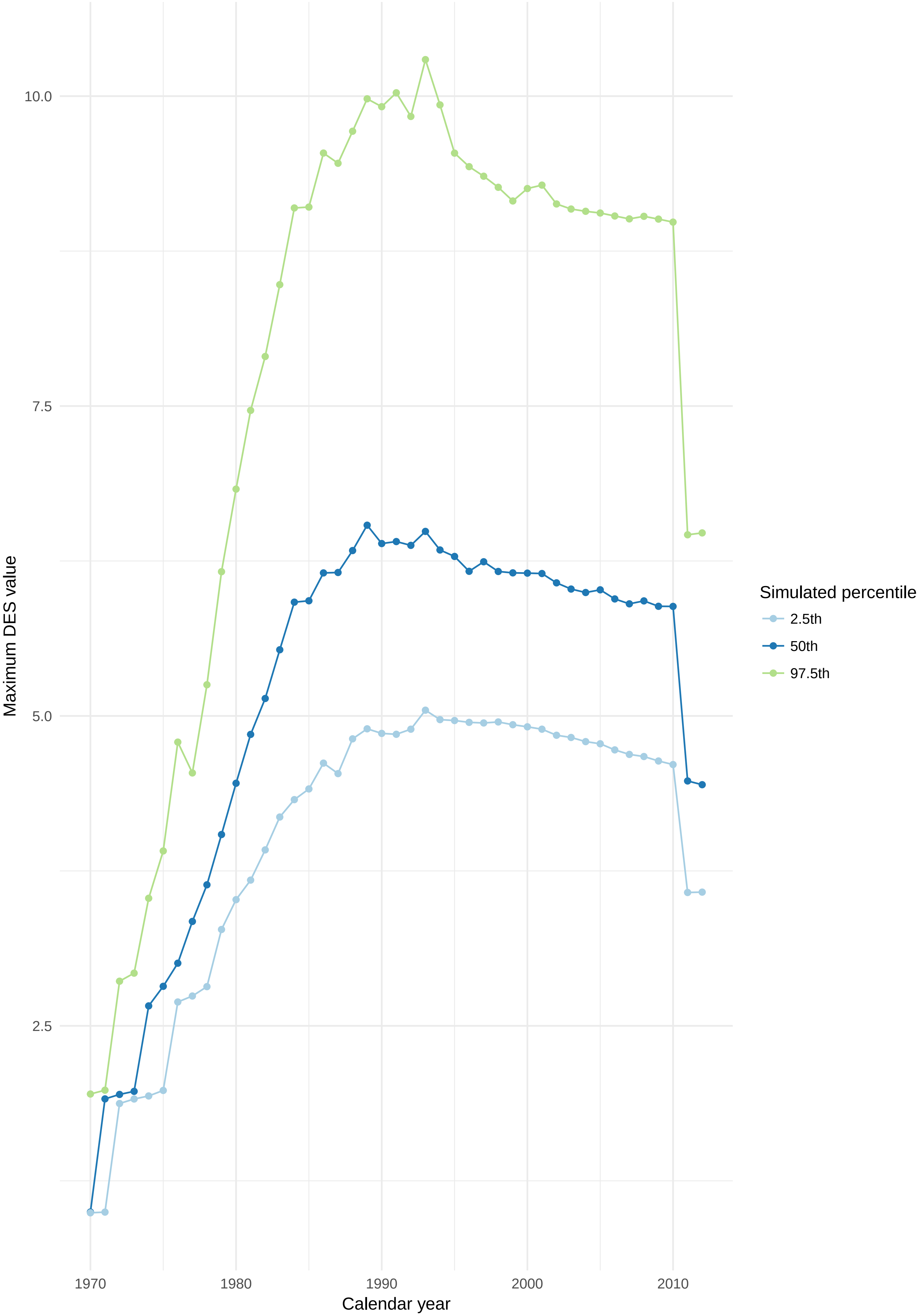

Maximum DES value
10.0
7.5
5.0
2.5
1970
1980
1990
2000
2010
Calendar year
Simulated percentile
2.5th
50th
97.5th

Supplementary Table 1. Relative risks of hepatitis B in relation to occurrence of the same disease in the contributing blood donor(s), presented overall and by latency in the donors

Disease	Donor diagnosed		Donor not diagnosed	
	Events/ person-years	Hazard ratio (95% CI)	Events/ person-years	Hazard ratio (95% CI)
a) Patients transfused before 1992				
All recipients transfused before 1992	1/11,699	1.2(0.2-8.3)	270/4,919,966	1.00 (ref)
<5 year latency in donor	0/964	0.0(0.0-n.e.)		
5-10 year latency in donor	0/2,000	0.0(0.0-n.e.)	270/4,919,966	1.00 (ref)
>10 years latency in donor	1/8,724	1.6(0.2-11.3)		
b) Patients transfused 1992-1996				
All recipients transfused 199-1996	1/3,067	1.6(0.2-11.3)	174/1,922,804	1.00 (ref)
<5 year latency in donor	1/1,097	4.4(0.6-32.3)		
5-10 year latency in donor	0/818	0.0(0.0-ne)	174/1,922,804	1.00 (ref)
>10 years latency in donor	0/1,152	0.0(0.0-ne)		
c) Patients transfused after 1996				
All recipients transfused after 1996	0/4,488	0.0(0.0-ne)	324/5,217,289	1.00 (ref)
<5 year latency in donor	0/2,153	0.0(0.0-ne)		
5-10 year latency in donor	0/1,512	0.0(0.0-ne)	324/5,217,289	1.00 (ref)
>10 years latency in donor	0/822	0.0(0.0-ne)		

Supplementary Table 2. Relative risks of Hepatitis B in relation to the maximum disease excess score among all contributing blood donors, presented stratified by calendar period of transfusion.

	**a) Before 1992**		**b) 1992-1996**		**c) After 1996**	
**Maximum disease excess score among contributing blood donors, categorized based on thresholds from simulated distribution***	Events/ person-years	Hazard ratio (95% CI)	Events/ person-years	Hazard ratio (95% CI)	Events/ person-years	Hazard ratio (95% CI)
<0	96/2,040,614	1.0(ref)	85/1,081,185	1.0(ref)	173/3,491,034	1.0(ref)
<2.5th percentile	170/2,871,984	1.3(1.0-1.8)	90/843,860	1.1(0.8-1.5)	150/1,728,870	1.3(1.0-1.6)
2.5th-50th percentile	3/11,293	4.2(1.3-14.1)	0/621	0.0(0.0-ne)	0/1,203	0.0(0.0-ne)
50th-97.5th percentile	2/6,450	5.5(1.3-23.1)	0/175	0.0(0.0-ne)	0/650	0.0(0.0-ne)
>97.5th percentile + donor not diagnosed	0/1,313	0.0(0.0-ne)	0/30	0.0(0.0-ne)	0/19	0.0(0.0-ne)
97.5th percentile + donor diagnosed	0/0	0.0(0.0-ne)	0/0	0.0(0.0-ne)	0/0	0.0(0.0-ne)
